# A New Early Finding of Moyamoya-Like Phenomena

**DOI:** 10.14740/jocmr2193w

**Published:** 2015-06-09

**Authors:** Waqas Jehangir, Zorawar Singh, Harsh Bhatt, Hasan F. Al-Azzawi, Kebir H. Bedran, Eric J. Uhrik, Abdalla Yousif, Teena Mathew

**Affiliations:** a Raritan Bay Medical Center, Perth Amboy, NJ, USA; b Ross University School of Medicine, Dominica

**Keywords:** Moyamoya disease, Graves’ disease, Leukocytosis

## Abstract

Moyamoya disease (MMD) primarily causes constriction of internal carotid artery, but it is known to extend to the middle and anterior cerebral arteries. Some of the symptoms caused by MMD include transient ischemic attack (TIA) and seizures. The etiology of MMD from Graves’ disease (GD) is mostly caused by thyrotoxicosis, but our finding of leukocytosis indicates a new finding that may help physicians prepare for the pending outcome of MMD from GD with leukocytosis. A 26-year-old Hispanic woman with a significant past medical history of GD and hypertension presented to the emergency department complaining of cough and shortness of breath for the past 5 days. During the patient’s stay in the hospital, the patient suddenly developed numbness of the right arm with subsequent right arm weakness 10 minutes later. The head CT showed no acute intercranial hemorrhage, but MRA showed right intracranial internal carotid artery stenosis, with marked focal stenosis along the proximal supraclinoid segment, moderate stenosis along its distal supraclinoid segment with marked stenosis along the origin of the right A1 segment. It was therefore believed to be moyamoya-like phenomena. We discuss an atypical presentation that led to moyamoya-like phenomena which we believe has not been described before. We believe that presentation of idiopathic leukocytosis may have triggered the cerebral stenosis.

## Introduction

Moyamoya disease (MMD) is a rare vascular disease that is not completely understood, but is seen in both the adult and adolescent population. An estimated 6% of strokes in childhood are caused by MMD [1]. The disease was first described by the Japanese as a “puff of smoke” largely due to the appearance on the angiogram. It was therefore assumed to largely affect the Asian population, but now is being seen in all ethic groups. MMD is characterized by progressive occlusion of the terminal portion of the internal carotid artery associated with the formation of collateral abnormal vessels at the base of the brain [1]. It is important to note that occlusions are generally bilateral, but unilateral occlusions do not rule out the condition. The disease is thought to be a result of intimal thickening, and subsequent formation of numerous anastomotic channels around the occluded vessels [2]. Interestingly, there is an association with Graves’ disease (GD) either before the diagnosis of MMD or afterwards [3]. The exact etiology of MMD is not well known, but since 10% of Japanese patient have a familial history of MMD, hereditary factors may appear to be involved.

## Case Report

A 26-year-old Hispanic woman, with a significant past medical history of GD and hypertension, presented to the emergency department complaining of cough and shortness of breath for the past 5 days. Her cough had progressively worsened over this time, as it became productive with bouts of blood mixed in the sputum. She denied any chest pain, dizziness, fever, chills, or any exposure to tuberculosis in the past.

She was tachycardic with a rate of 103/min, hypertensive with a blood pressure of 153/121 mm Hg and tachypneic with a respiratory rate of 30/min. Her oxygen saturation was 99% on 2 L nasal cannula and afebrile. Upon physical examination, the only significant findings were decreased bibasilar bilateral air entry and bilateral crackles moderately more intense on the right side. Only noteworthy labs showed a white blood cell (WBC) count of 15.3 cells/mm^3^ and a brain natriuetic peptide (BNP) of 11,715.

**Figure 1:**
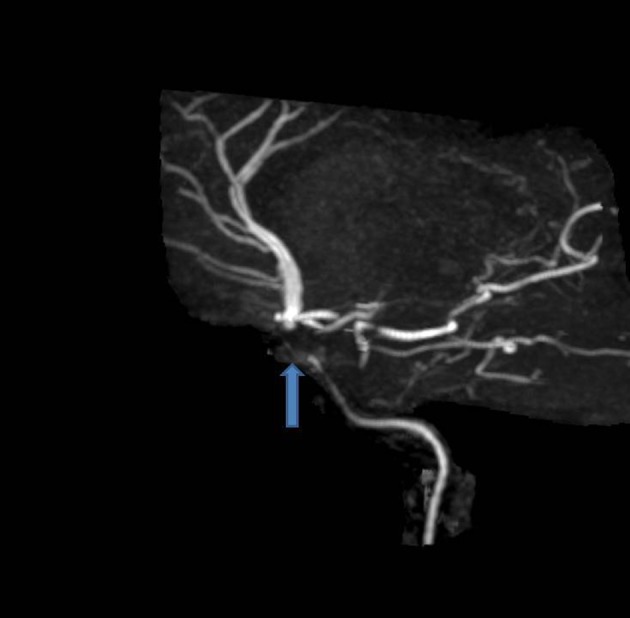
MRA head showing a right intracranial internal carotid artery stenosis, with marked focal stenosis along the proximal supraclinoid segment, moderate stenosis along its distal supraclinoid segment with marked stenosis along the origin of the right A1 segment.

**Figure 2:**
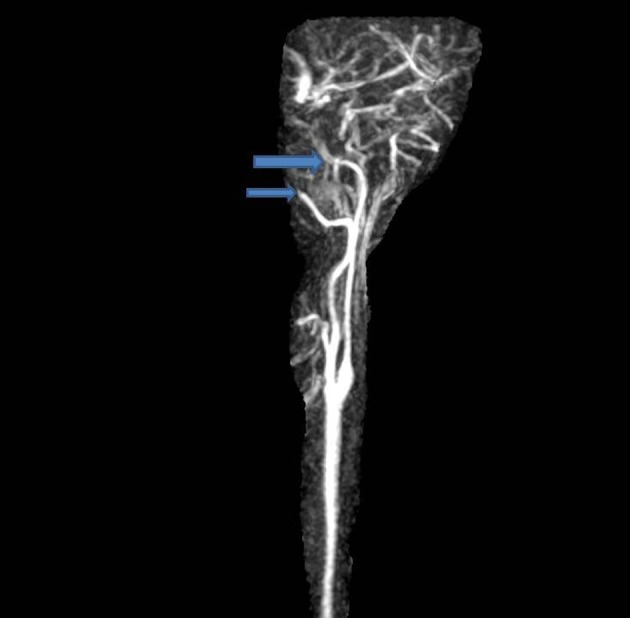
MRA neck showing diffuse decreased caliber of the cervical left internal carotid artery.

The patient was admitted into the hospital for further pulmonary testing. During the patient’s stay in the hospital, the patient suddenly developed numbness of the right arm with subsequent right arm weakness 10 min later. A right facial droop accompanied with slurred speech was noticed. A code stroke was initiated and a stat head CT showed no acute intercranial hemorrhage and stat EKG showed a sinus rhythm. MRA of head showed a right intracranial internal carotid artery stenosis, with marked focal stenosis along the proximal supraclinoid segment, moderate stenosis along its distal supraclinoid segment with marked stenosis along the origin of the right A1 segment (Fig. 1). Furthermore, there was a marked stenosis of the left intracranial internal carotid artery along its petrous, cavernous and proximal supraclinoid segments. Multiple stenoses along segments of both posterior cerebral arteries, both superior cerebellar arteries, both anterior inferior cerebral arteries, and the left posterior interior cerebellar artery were noticed as well. MRA of neck showed diffuse decreased caliber of the cervical left internal carotid artery (Fig. 2). From these significant findings, we can see that this case presents similar to patients with MMD. Tissue plasminogen activator (tPA) was administered to the patient, which initially improved her symptoms temporarily, but was not a permanent solution. The decision to transfer the patient for further neurological management was made by the house neurologist and the patient will be followed up and managed at that center. Further workup at the center included checking for vasculitis, which was negative.

## Discussion

MMD is a devastating condition that is responsible for abrupt stroke like symptoms, headaches, seizures, and learning impairment. MMD is known to occur with some conditions such as sickle cell disease, cerebral arterial-venous malformation, neurofibromatosis, and prior brain surgery.

There have been numerous studies about the relationship between MMD and GD; however, the exact mechanism causing MMD from GD is not fully understood. Our patient’s past medical history of GD raises a concern for a likely reason for the etiology for MMD and MMD-like symptoms. One hypothesis is that an increase in thyroid antibodies triggers the sympathetic norepinephrine containing nerve fibers. This then results in vasoconstriction of intracranial arteries [2].

Another study examined the relationship between the thyrotoxicosis and MMD. In a state of toxicosis, increasing production of thyroid hormone increases cerebral metabolism and thus increases the oxygen consumption [3]. In patients who already have GD, this may greatly increase the occurrence of MMD. In addition, thyrotoxicosis is known to cause a hypercoagulable state, which will also cause ischemia. It is important to note that thyrotoxicosis is the trigger for a majority of the patients who had MMD accompanied with GD [3]. Some of the important symptoms of thyrotoxicosis include anxiety, confusion, diarrhea, palpitations, dyspnea, chest pain, and diplopia [4]. Interestingly, our patient did not meet the criteria for thyrotoxicosis.

Our patient atypically progressed from GD to MMD in a way that has not been described before to our knowledge. There is evidence suggesting that autoimmune diseases, other than GD, may also trigger MMD. Some of these autoimmune diseases include antiphospholipid antibodies syndrome, systemic lupus erythematous, HLA class I or II alleles abnormality [5]. Our patient did not have any other autoimmune conditions except for GD.

The patient’s high WBC count may have indicated a new trigger to vasoconstriction of the cerebral arteries. New studies have shown that some infections have preceded MMD. Some of these infections are bacterial meningitis, tuberous infections, varicella-zoster virus, and measles virus [5]. At the time of admission, the patient was afebrile and did not show any signs of infection. This leaves us with a puzzling question, what could have induced a cerebral vasoconstriction in our patient?

There have been studies showing that there is leukocytosis present with unstable angina pectoris and acute myocardial infarction. Although the mechanism is not fully understood *in vitro* and *in vivo* experiments have shown that activated leukocytes have resulted in vasoconstriction [6]. This leads us to believe that the idiopathic cause of leukocytosis and the patient’s past medical history of GD may have led to MMD.

### Conclusion

MMD is caused by stenosis of the cerebral arteries. It is a progressive disease that was initially thought to be predominant in the Japanese population, but is now seen in all ethnic groups. GD is found to have a robust connection with MMD. The etiology of MMD from GD is mostly caused by thyrotoxicosis, but our finding of leukocytosis indicates a new finding that may help physicians prepare for the outcome of MMD or MMD-like symptoms in patients with GD presenting with an increase in WBC count.
